# Movement intensity demands between training activities and competition for elite female netballers

**DOI:** 10.1371/journal.pone.0249679

**Published:** 2021-04-07

**Authors:** Edward R. Brooks, Amanda C. Benson, Aaron S. Fox, Lyndell M. Bruce

**Affiliations:** 1 Centre for Sport Research, School of Exercise and Nutrition Sciences, Deakin University, Burwood, Victoria, Australia; 2 Department of Health and Medical Sciences, Sport Innovation Research Group, Swinburne University of Technology, Hawthorn, Victoria, Australia; Universidade Federal de Minas Gerais, BRAZIL

## Abstract

The aim of this study was to assess the differences in movement intensity demands between training activities and competition match-play in elite netball. Twelve elite female netballers (mean ± SD, age = 25.9 ± 5.1 years; height = 178.6 ± 8.9 cm, body mass = 71.1 ± 7.1 kg) competing in Australia’s premier domestic netball competition participated. Data were collected across the season from all pre-season training sessions (*n* = 29), pre-season practice matches (*n* = 8), in-season training sessions (*n* = 21), in-season practice matches (*n* = 5), and competition matches (*n* = 15). Linear mixed-effects models assessed differences in PlayerLoad™ per minute and metreage per minute between activity types (Specialist, Skill Drills, Set-piece, Match Scenarios, Practice Match-play, and Competition Match-play) for positional groupings (Defenders, Midcourters, and Goalers). Competition Match-play resulted in higher (p < 0.05) PlayerLoad™ than all training activity types, with the largest magnitudes of difference between Specialist–Competition (*d* = 0.44–0.59; small to medium) and Skill Drills–Competition (*d* = 0.35–0.63; small to medium) for all positional groups. The smallest difference was found between Match Scenarios–Competition (*d* = 0.12–0.20; trivial to small) and Practice Match-play–Competition (*d* = 0.12–0.14; trivial). Competition Match-play also resulted in higher (p < 0.05) metreage per minute than Specialist (*d* = 0.23–0.53; small to medium), Skill Drills (*d* = 0.19–0.61; trivial to medium) and Set-piece (*d* = 0.05–0.31; trivial to small). Training activity demands in order of least to most similar to competition were specialist, skill drills, set-piece, match scenarios, and practice match-play. We provide data that enables coaches and physical preparation staff to incorporate progressions into their training session designs that can replicate the movement intensity demands of competition in training.

## Introduction

It is commonly asserted in team-sport coaching and physical preparation literature that training should provide opportunities for athletes to experience the demands of match-play prior to competition [1–3]. Drills and training activities with fewer similarities to the demands and constraints of competition may not adequately reflect the physiological demands and movement patterns required in match-play; however, they may still provide specific technical or tactical development [4]. A multifactorial approach to team sport periodisation may optimise performance, especially where these factors can be integrated into a cohesive season structure or cycle [5]. One approach often undertaken by practitioners is to move towards training activities that are more reflective of competition performance.

Young et al. [6] were the first to compare training to match demands in elite netball, finding that training sessions lasted longer and accumulated more total PlayerLoad™ than matches. However, the intensity of movement (relative PlayerLoad™; au·min-^1^) was higher during match-play [6]. It is unclear whether different activity types within training produced higher intensity movements as sessions were not separated into activity types or coded to remove breaks between drills [6]. While there is a lack of published data exploring the external load of specific drills or activities within training sessions in elite netball, some preliminary analysis has been done using a sub-elite population [7].

Analysis of accelerometer-derived training and match loads in a sub-elite netball population have shown that movement intensity, as measured by PlayerLoad™ per minute, increased from skills to game-based training (6.0 to 9.0 au·min-^1^) [7]. However, it appears that sub-elite netballers perform at a lower intensity during match-play, than their elite counterparts [7, 8]. This is also supported by analysis comparing recreational netballers to sub-elite netballers; showing higher movement intensity for the higher-standard players (sub-elite; 9.96 ± 2.50 au·min-^1^) compared to the lower-standard players (recreational; 6.88 ± 1.88 au·min-^1^) [9]. Chandler et al. [7] highlight the importance of quantifying differences in movement intensity across training drills and match-play in these sub-elite players; however, all court-based skill drills were classified into either skills training (e.g., passing, catching, and movement patterns) or game-based training (e.g., match-play with reduced players, increased court area, and rule changes) categories. While some additional categories were included for specific conditioning tasks [7], this may not differentiate between the full range of on-court, coach-prescribed, activities involved in netball training and may limit the specificity of training prescription. An improved understanding of the movement intensity demands of specific training activities would allow coaches to refine session designs to emphasise relevant activities that suit the stage of the season, or athlete management needs (e.g., opt to either increase the proportion of high-volume, low-intensity activities or low-volume, high-intensity activities). Objectively assessing a greater number of categories of common netball training activities will allow coaches to make adjustments with greater precision and confidence.

A comprehensive analysis categorising drills into a range of commonly prescribed activity types in elite netball training would provide the missing detail and further elucidate which tasks are most representative of competition match-play and inform training programme prescription in elite netball. It is therefore important to explore whether elite netballers are being exposed to the demands of competition during any training activities, as integration of skill and physical movement demands may provide an optimised approach for coaches to periodise athletes during return from injury, or in preparation for competition match-play. The current study aims to compare the movement intensity demands of different activity types which occur in training to those in competition match-play in elite netball.

## Materials and methods

### Participants

Twelve elite female netballers (mean ± SD, age = 25.9 ± 5.1 years; height = 178.6 ± 8.9 cm, body mass = 71.1 ± 7.1 kg) covering the following positional groupings; Goalers (Goal Shooter, Goal Attack; *n* = 3), Defenders (Goal Keeper, Goal Defender; *n* = 4), and Midcourters (Centre, Wing Attack, Wing Defence; *n* = 5) agreed to participate in the current study. The participants recruited for this study all competed in Australia’s premier netball competition (Suncorp Super Netball) during the 2019 season. Data were collected across the pre-season, in-season, and finals phases of the 2019 season. An extended pre-season occurred over 15 weeks, commencing in early January. Competition matches were scheduled weekly over the 14 rounds of regular-season competition, with a scheduled break occurring between round 9 (23^rd^ June) and round 10 (27^th^ July) to accommodate the quadrennial Netball World Cup. All participants are considered to be semi-professional or professional athletes and were contracted with an elite club during data collection. Athletes provided written informed consent prior to commencing their participation in this study. Ethical approval was granted by the Deakin University Human Ethics Advisory Group (approval code: HEAG-H 206_2018).

### Data collection

A longitudinal single-cohort observational research design was used. Data collection occurred over a full-season of elite netball (pre-season and competitive season). Data were recorded and included from all coach-prescribed, on-court, pre-season training sessions (*n* = 29), pre-season practice matches (*n* = 8), in-season training sessions (*n* = 21), in-season practice matches (*n* = 5), and competition matches (*n* = 15). All off-court training including strength and conditioning training were not assessed in this study. Meterage per minute was collected at all training sessions; however, only one competition stadium was equipped with the required local positioning system nodes, which limited the sample to six competition matches. Pre-season tournament matches (*n* = 4) was excluded from this analysis due to the differences in structure (i.e. 10-minute quarters) and rules (e.g. rolling-substitutions) when compared to regular-season competition match-play. Every activity the athletes participated in during training was categorised to represent how closely the drill represented competition and were classified as;

- *Specialist*—Technical and tactical drills where position-specific tasks were performed in small groups with a high level of coach interaction (e.g., Midcourters feeding, Goalers shooting, and Defenders circle movement).
- *Skill Drills*—Ball control and passing drills which can be basic or complex and vary in movement requirements (e.g., high-repetition passing with planned movement patterns).
- *Set-piece—*Highly structured drills where players are in defined court positions and play out set-plays or test structures (e.g., playing out a ball from a stoppage to goal).
- *Match Scenarios—*Simulated match scenarios where players are in defined court positions without strictly instructed plays, however, match constraints such as time-limits and scoring are imposed (e.g., ‘Team A’ has a two-goal deficit with 30 seconds of play remaining, ‘Team B’ starts with possession).
- *Practice Match-play*—Players are in defined court positions and regular match constraints are imposed; however, coaches may alter the length of quarters or change athletes between teams (e.g., 10-minute quarters with player and position changes within and/or across teams at each quarter break).

Categories were determined after consultation with three accredited and experienced netball coaches working for a Suncorp Super Netball club and covered all possible prescriptions of on court coach-led training tasks. Competition match-play activities comprised of league sanctioned, regulated, and officiated match-play occurring during the season.

Participants wore a Catapult T6 unit in a fitted sport vest, with the devices mounted in a pouch on their upper-back. Each device contained a 10Hz Local Positioning System (LPS) tag and 100Hz inertial sensors (tri-axial accelerometer, gyroscope and magnetometer). These wearable devices were wirelessly connected to the ClearSky LPS (Catapult Sports, Melbourne, Australia) using ultra-wideband communication and tracked positional movement and inertial changes for all athletes simultaneously. The ClearSky LPS consisted of a pre-installed network of anchors within the training stadium and home competition stadium. The ClearSky LPS has acceptable validity for time-motion analysis in team sports which are played in an indoor environment [10]. Additionally, accelerometer-derived measures, including PlayerLoad™ and its associated variables, have shown good reliability and sensitivity for use in indoor team sports [11]. PlayerLoad™ per minute from all sessions and metreage per minute from venues with LPS capabilities were selected as the variables of focus for this study. PlayerLoad™ is defined as the “sum of instantaneous rate of change in acceleration (jerk) across the three planes (x, y and z), divided by a scaling factor (100)” [8]. Metreage per minute is defined as the “accumulated distance in metres across the specified period divided by the duration in minutes” [8]. These provide relative measures (divided by time) of accelerometer-derived total movement intensity and running specific intensity derived from a local positioning system [8].

Openfield software (v1.22.0) was used live throughout all training sessions and matches to monitor and code activity types. Training sessions were coded live to ensure coaching stops and inherent breaks between drill blocks were not included in the activity analysis. However, planned rest intervals specified by the coaches within drills (e.g., players rotating at set intervals, or drills with an inherent active and recovery component) were included. Bench-time and breaks between quarters were excluded from all match-play data (practice matches and competition matches). At the end of the season individual athlete activity data were extracted from their respective sessions into the aforementioned six categories; specialist (*n* = 230), skill drills (*n* = 1509), set-piece (*n* = 752), match scenarios (*n* = 167), practice match-play (*n* = 401), or competition match-play (*n* = 461). A total of 3520 unique activity sessions across all athletes and session types over the course of the full season were included for analysis.

### Data analysis

Linear mixed-effects models were run for PlayerLoad™ per minute and metreage per minute to compare each activity type to competition match-play. These models are a versatile class of statistical model commonly used to assess longitudinal data sets, as they can be used to characterise and compare changes in a specified variable of interest [12]. Furthermore, they can deal with the challenges around data dependency and missing data which can occur due to injury, illness or performance exclusion. The statistical computing program R was used for data analysis [13]. A separate linear mixed-effects model was produced for each variable (PlayerLoad™ and metreage per minute) using the ‘lmerTest’ package (v3.1–2) [14]. Models were fitted using restricted maximum likelihood (REML) and p-values were derived using Kenward-Roger approximated degrees of freedom [15]. The alpha level was set at 0.05. The inputs to the model’s formula were activity type, positional grouping, and their interaction (fixed effects), along with player and week during the season (random effects). The model included player as a random effect to recognise the influence of each individual on the group differences. Model comparisons, contrasts, and post-hoc multiplicity adjustments (Tukey method) were performed using the ‘emmeans’ package (v1.4.7) [16]. Contrasts were calculated for both activity type and position; however, only activity type contrasts are reported in this paper as this was the primary research aim (see S1 File for positional contrasts). Effect sizes (Cohen’s *d*) [17] for the contrasts, along with their respective 95% confidence intervals, were calculated using the ‘effectsize’ package (v0.3.1.1) [18]. Effect sizes were interpreted as small (*d* = 0.2), medium (*d* = 0.5), and large (*d* = 0.8) [17]. Plots were produced using the ‘ggplot2’ package (v3.3.2) [19].

## Results

**Fig 1:**
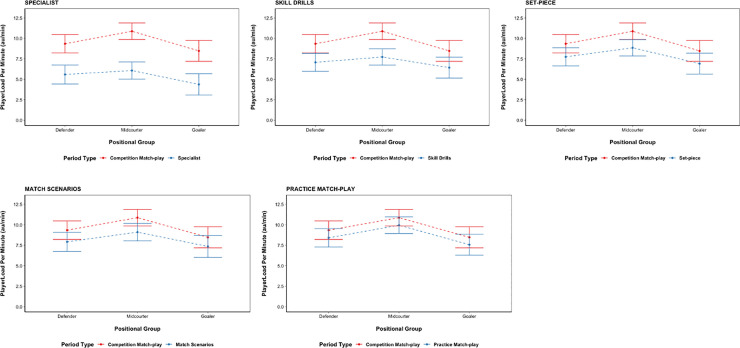
Comparison of PlayerLoad™ per minute for each activity type against competition Match-play. The model estimated marginal means and 95% confidence intervals are presented for each of the comparisons. Abbreviations; au/min = arbitrary units per minute.

Statistically significant differences (p < 0.05) were found between all training activity types and Competition Match-play for the PlayerLoad™ per minute variable, with Competition Match-play resulting in the highest PlayerLoad per minute across all activity types (see Table 1). The largest magnitudes of difference in PlayerLoad™ per minute between activity types for all positional groups were; Specialist–Competition Match-play (*d* = 0.44–0.59; small to medium) and Skill Drills–Competition Match-play (*d* = 0.35–0.63; small to medium), based on their effect size. The smallest magnitudes of differences in PlayerLoad™ per minute were found to be between Match Scenarios–Competition Match-play (*d* = 0.12–0.20; trivial to small) and Practice Match-play–Competition Match-play (*d* = 0.12–0.14; trivial) (see Fig 1).

**Table 1:** Model contrasts comparing each training activity type to competition Match-play by positional group.

Positional Grouping	Contrast	Estimate	*SE*	*df*	*t* ratio	Cohen’s *d*	Cohen’s *d* 95% CI	p value
**PlayerLoad™ per minute**								
**Defender**	Competition Match-play—Practice Match-play	0.93	0.24	3488.16	3.84	0.13	0.06–0.20	< 0.01
	Competition Match-play—Match Scenarios	1.43	0.31	3494.82	4.64	0.16	0.09–0.22	< 0.01
	Competition Match-play—Set-piece	1.59	0.21	3494.98	7.56	0.26	0.19–0.32	< 0.01
	Competition Match-play—Skill Drills	2.27	0.20	3489.90	11.47	0.39	0.32–0.46	< 0.01
	Competition Match-play—Specialist	3.75	0.29	3494.36	12.97	0.44	0.37–0.51	< 0.01
**Midcourter**	Competition Match-play—Practice Match-play	0.92	0.22	3457.05	4.16	0.14	0.07–0.21	< 0.01
	Competition Match-play—Match Scenarios	1.77	0.29	3492.45	6.04	0.20	0.14–0.27	< 0.01
	Competition Match-play—Set-piece	2.02	0.18	3492.63	10.96	0.37	0.30–0.44	< 0.01
	Competition Match-play—Skill Drills	3.14	0.17	3453.12	18.48	0.63	0.56–0.70	< 0.01
	Competition Match-play—Specialist	4.80	0.28	3488.97	17.41	0.59	0.52–0.66	< 0.01
**Goaler**	Competition Match-play—Practice Match-play	0.90	0.25	3487.65	3.62	0.12	0.06–0.19	< 0.01
	Competition Match-play—Match Scenarios	1.12	0.33	3493.38	3.40	0.12	0.05–0.18	0.01
	Competition Match-play—Set-piece	1.56	0.21	3493.34	7.36	0.25	0.18–0.32	< 0.01
	Competition Match-play—Skill Drills	2.04	0.20	3485.95	10.38	0.35	0.28–0.42	< 0.01
	Competition Match-play—Specialist	4.10	0.27	3493.14	14.96	0.51	0.44–0.57	< 0.01
**Metreage per minute**								
**Defender**	Competition Match-play—Practice Match-play	-1.18	3.11	2930.67	-0.38	-0.01	-0.09–0.06	1.00
	Competition Match-play—Match Scenarios	5.48	3.42	2928.60	1.60	0.06	-0.01–0.13	0.60
	Competition Match-play—Set-piece	8.70	2.70	2937.72	3.22	0.12	0.05–0.19	0.02
	Competition Match-play—Skill Drills	18.40	2.64	2930.18	6.98	0.26	0.19–0.33	< 0.01
	Competition Match-play—Specialist	26.86	3.24	2930.20	8.29	0.31	0.23–0.38	< 0.01
**Midcourter**	Competition Match-play—Practice Match-play	-0.24	2.70	2915.97	-0.09	0.00	-0.08–0.07	1.00
	Competition Match-play—Match Scenarios	10.77	3.08	2918.26	3.49	0.13	0.06–0.20	0.01
	Competition Match-play—Set-piece	14.55	2.27	2931.10	6.41	0.24	0.16–0.31	0.01
	Competition Match-play—Skill Drills	31.69	2.18	2901.08	14.50	0.54	0.46–0.61	< 0.01
	Competition Match-play—Specialist	36.23	2.92	2918.83	12.42	0.46	0.39–0.53	< 0.01
**Goaler**	Competition Match-play—Practice Match-play	2.41	3.24	2934.91	0.74	0.03	-0.04–0.10	0.98
	Competition Match-play—Match Scenarios	8.49	3.59	2933.67	2.36	0.09	0.01–0.16	0.17
	Competition Match-play—Set-piece	11.72	2.73	2937.07	4.29	0.16	0.09–0.23	< 0.01
	Competition Match-play—Skill Drills	20.66	2.66	2931.13	7.78	0.29	0.21–0.36	< 0.01
	Competition Match-play—Specialist	33.49	3.15	2930.61	10.64	0.39	0.32–0.47	< 0.01

Abbreviations: SE, Standard Error; df, Degrees of Freedom; Defender: Goal Defence, Goal Keeper; Midcourter: Centre, Wing Attack, Wing Defence; Goaler: Goal Shooter, Goal Attack.

**Fig 2:**
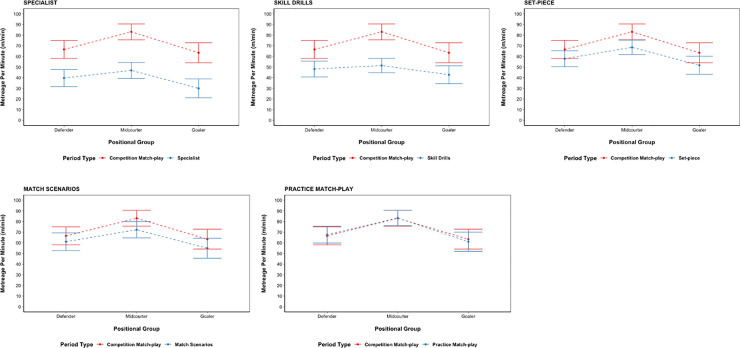
Comparison of Metreage per minute for each activity type against competition Match-play. The model estimated marginal means and 95% confidence intervals are presented for each of the comparisons. Abbreviations; au/min = arbitrary units per minute.

When using the metreage per minute variable, statistically significant differences (p < 0.05) were found between Competition Match-play and Specialist, Skill Drills and Set-piece for all positional groups. In each case, Competition Match-play resulted in higher metreage per minute. No significant differences in meterage per minute were found for any positional group when comparing Practice Match-play–Competition Match-play (p > 0.05). Differences varied between positional groups, with the largest differences in Metreage per minute between activity types for Defenders and Goalers being; Specialist–Competition Match-play (*d* = 0.31–0.39; small) and Skill Drills–Competition Match-play (*d* = 0.26–0.29; small). The largest difference in Metreage per minute between activity types for Midcourters was for Skill Drills–Competition Match-play (*d* = 0.54; medium), followed by Specialist–Competition Match-play (*d* = 0.46; small). Midcourters were also found to have a statistically significant difference in meterage per minute between Match Scenarios–Competition Match-Play (p = 0.01, *d* = 0.13; trivial); however, no significant differences were found for Defenders or Goalers (p > 0.05) (see Fig 2).

## Discussion

Our study presents a direct comparison between different training activity types and competition match-play using two movement intensity variables (PlayerLoad™ and metreage per minute) to assess whether the players were exposed to similar movement intensity demands during any components of training. The results of this analysis reveal that the training activities, in order of least to most similarity to competition, are; specialist, skill drills, set-piece, match scenarios, and practice match-play. Understanding the demands of these common training activities allows for a periodised approach to be applied to both skill and physical movement demands in one integrated plan.

Across all positional groups (Goalers, Defenders, and Midcourters), PlayerLoad™ per minute for Practice Match-play closely represented Competition Match-play, whilst Specialist activities were the least representative. It is unsurprising that Practice Match-play was similar to Competition Match-play as they are played under the same conditions, usually with only minor variations, such as the length or number of the quarters played, which are largely accounted for when using relative measures (i.e. per minute). In the only other study to separate netball training activities into specific types, game-based training also resulted in increased movement intensity above standard skills components [7]. However, in contrast to the current study, the mean movement intensity surpassed match-play intensity during game-based training involving reduced player numbers, larger playing area, and rule changes [7]. This may be explained by the athletes in the sub-elite setting using larger areas with fewer opponents [7], whereas the elite-level may increase opponents and play on a half-court area when attempting to replicate high intensity match-play, as opposed to primarily increasing running demands.

Metreage per minute followed the general trends of PlayerLoad™ per minute, across all activities, with movement intensity demands differing to a larger extent for the closed drills or drills where speed was reduced and technique was the central focus (i.e., Specialist and Skill Drills). The main point of difference between the two variables was that no statistically significant differences were found in running demands (metreage per minute) between Practice Match-Play and Competition Match-play across all positional groups, despite a difference being found for PlayerLoad™. This may be due to the way in which these two variables measure intensity. Metreage per minute is a spatiotemporal measure that is collected using a local positioning system to determine positional co-ordinates on the court, which are then used to calculate the accumulated distance covered for the given time period [10]. In contrast, PlayerLoad™ is collected using a tri-axial accelerometer and sums the instantaneous rate of change in acceleration (jerks) in three planes (x, y, and z), divided by a scaling factor (100) [8]. The difference in findings between these measures may be a result of additional movements, such as jumps and bumps, which can be captured using PlayerLoad™, as opposed to distance-based measures alone (e.g., metreage per minute). Given that netball involves high jumping and change of direction demands [8], it may be preferable to use PlayerLoad™ in situations where only one variable is collected or used to represent external load. The differences between these variables may also be influenced by the smaller sample size of the metreage per minute variable, which was only captured in venues equipped with local positioning system nodes, although there were still 2,962 data points analysed in this model.

The movement intensity demands of Specialist activities are very different to those of Competition Match-play; however, these activities retain an important role in the context of technical skill performance [20]. Specialist activities often involve working on one aspect of performance that is critical to the positional group, and therefore other environmental aspects normally found in a match are removed to enable specialist technical skills to be developed. Examples include removing teammates and/or defenders to allow for systematic repetition of a skill with unencumbered execution, such as to practice goal shooting, which can result in drill design differing significantly from match-play. However, constant practice can be very tedious and lack challenge for elite performers, so variable (e.g., changing shooting distance or position) or random (e.g., shot, feed, feed, shot) practice of important skills may be preferable when non-representative activity types are incorporated [21].

A key priority for coaches is to design and implement training sessions that balance each athlete’s technical and tactical skill development with the physical demands and stimuli required to perform in competition. Coaches can use the findings of this study to structure training programs that can progress athletes from pre-season to competition or return to play from injury. This can be achieved by adjusting successive sessions to increase the time spent performing activities that better replicate competition intensity, as the athlete or season progresses (i.e., increasing match scenarios, set-piece, and/or practice match-play). Development coaches may also use this information to assist in the effective transition of their athletes to higher levels of the sport through progressively increasing the proportion of representative drills performed in training. This also allows for an agile and adaptable program where coaches can focus on any specific technical or tactical elements that arise as priorities throughout the season, while also progressing intensity and skills in a structured manner.

The results reported in this study use objectively quantified data to validate the assumption that increasing the similarity of the structure and content of the training activity types to Competition Match-play will lead to higher movement intensity demands. This allows coaches and support staff to better understand the magnitude of differences in movement intensity between each training activity and Competition Match-play, based on the effect sizes. This may subsequently allow for greater control of session intensity and allow coaches to confidently increase or decrease movement intensity in a more agile manner.

There are some important considerations and limitations of this study when interpreting these findings. The first is that while this is a large set of data across a full pre-season and season of elite netball, the data has only been collected on one elite netball team and may not be indicative of all other elite netball teams. Similarly, the number of players in each playing group were also limited by the restricted number of overall participants. Match data was not classified based on match outcome, to maintain anonymity of the players, therefore; it should not be used to infer quality of performance. This analysis was focussed on the training demands incurred during on-court sessions and did not include off-court activities. Additionally, positional groupings (Defenders, Midcourters, and Goalers) have been used for athlete classification, as most athletes played across more than one court-position during the course of a full season (but always within their positional grouping) and this classification method also protects the anonymity of the participants.

## Conclusion

This study shows a clear progression of training activities that can be implemented by coaches and physical preparation staff intending to replicate the physical movement demands of competition in training sessions, from specialist, skill drills, set-piece, match scenarios, to practice match-play. Specialist and skill drills do not replicate the movement intensity demands of competition; however, these may be important for coaching technical elements in netball. Coaches and physical preparation staff intending to replicate the movement intensity demands of competition in a training setting should incorporate practice match-play sessions into the season training schedule. Understanding the demands of common training activities allows for a periodised approach to be applied to both skill and physical movement demands in one integrated plan. Composition of training sessions can be progressively adjusted to transition an athlete, or team of athletes, from basic pre-season tasks to competitive match-play.

## Supporting information

S1 File Full linear mixed model contrasts. (XLS)
